# Quantifying Consumer Interest in Medicare Advantage: Development and Usability Study Using Google Trends Data

**DOI:** 10.2196/89355

**Published:** 2026-03-27

**Authors:** Amy Dunn Tramontozzi, Gregory J Downing, Lucas Tramontozzi

**Affiliations:** 1 Data Science Institute University of Chicago Chicago, IL United States; 2 School of Health Georgetown University Washington, DC United States; 3 Innovation Horizons Washington, DC United States

**Keywords:** Medicare Advantage, health plan marketing, consumer behavior, health policy, Google Trends, time-series analysis, digital health surveillance

## Abstract

**Background:**

Since 2020, Medicare Advantage (MA)–related internet searches have tripled, accompanied by increased regional marketing by private insurers. Commercial health insurance dominates the internet during enrollment periods, often outpacing public sources in accessibility. Prior studies suggest that MA advertising significantly shapes enrollment and may fuel choices over traditional Medicare in certain subpopulations. We sought to better understand how health plan marketing strategies affect consumers by using Google Trends data and MA health plan enrollment selection. We applied novel analysis to assess statistical relationships among marketing, internet searches, and enrollment data.

**Objective:**

The objectives of this paper are (1) to establish the validity of Google Trends data as a surrogate measure for consumer MA plan selection by demonstrating stable, repeatable seasonality and domain specificity using control terms such as “car insurance” and “life insurance” at national and Designated Market Area levels; (2) to quantify the congruency between MA search interest and Centers for Medicare & Medicaid Services enrollment data by testing whether search peaks coincide with or precede enrollment surges nationally within a year; and (3) to assess whether local search intensity aligns with advertising exposure by evaluating search behavior as a potential proxy for marketing impact and consumer engagement.

**Methods:**

This study is a retrospective Google Trends analysis of consumer search patterns from January 2004 to December 2024, using relative search volume and conducting correlations with MA enrollment. Search data are accessible via the Google Trends website Explore tool or by applying for Google Trends application programming interface alpha access. MA enrollment data originated from the Centers for Medicare & Medicaid Services MA Dashboard. KFF (formerly the Kaiser Family Foundation) provided the medical advertising marketing data.

**Results:**

A consistent, significant correlation between MA advertising and searches on MA exists across US markets, particularly before and during MA enrollment windows. Findings suggest a linkage in user behavior between volume of searches and subsequent enrollment in an MA plan.

**Conclusions:**

Internet search data can provide an open, near-real-time means of tracking patterns in MA-related search activity across time and geography, offering insight into how consumer interest fluctuates around enrollment periods. Our analysis reveals repeatable patterns in consumer interest over time that may be useful for contextualizing insurance marketing dynamics of consumers choosing commercial MA over traditional Medicare benefits. We also identified a significant correlation of seasonal trends in searches using terms associated with MA plans that peaked during the annual enrollment period (October-December). Improved accessibility to Medicare resources and directed messaging can bridge information gaps for underserved populations and can lead to more cost-effective decision-making by Medicare-eligible beneficiaries.

## Introduction

### Background

In the United States, individuals who reach 65 years of age or who otherwise qualify through disability or chronic illness may be eligible for Medicare, a federal health care benefits program that provides coverage through multiple parts and options. Among these, Medicare Advantage (MA), also known as Part C Medicare, consists of privately administered, capitated health plans that serve as an alternative to traditional (Original) Medicare [1]. MA plans receive risk-adjusted payments from the Centers for Medicare & Medicaid Services (CMS) and are permitted to offer supplemental benefits that are not available in Original Medicare, such as dental, vision, hearing, and fitness services. Unlike traditional Medicare benefits, commercial MA plans are not required to provide CMS with cost-of-care data, can create provider networks of negotiated rates and performance characteristics, and require beneficiary copays for services and other out-of-pocket expenses. Enrollment in MA has expanded rapidly over the past decade; as of 2025, more than half of all Medicare beneficiaries are enrolled in MA plans administered by commercial health plans [2]. This growth has been accompanied by substantial increases in federal spending, with per-beneficiary payments estimated at 122% of comparable spending under Original Medicare—equating to roughly US $83 billion in additional annual outlays [3]. Past evidence has suggested that consumers may not easily understand the distinctions among benefit plans that can lead to making less cost-effective choices, and this may particularly be the case for individuals with chronic conditions who require more high-cost care and for individuals of low income status [4].

### Marketing, Consumer Choice, and Information Asymmetry

Although the MA program has introduced flexibility and supplemental benefits for many beneficiaries, its expansion has also raised concerns regarding how consumers navigate plan choices and how marketing shapes those decisions.

A patient’s choice among MA plans may have significant financial and medical implications, and choices abound—here are thousands of MA plans available that differ in benefit design, care coordination, and physician networks [5]. However, the overall trend is that MA plans are becoming increasingly deficient in their coverage of enrollees’ needs. For example, the American Hospital Association found that denials of coverage by MA plans rose approximately 56% between 2022 and 2023 [6]. Such growing deficiencies are bolstered by a lack of accountability on the part of regulatory authorities. A 2022 audit by the Office of the Inspector General found that MA plans “sometimes delayed or denied Medicare Advantage beneficiaries’ access to services, even though the requests met Medicare coverage rules” [7]. Reflecting the magnitude of this problem for consumers, both the Office of the Inspector General and the US Government Accountability Office have urged policymakers to strengthen auditing of MA insurers, but policymakers themselves have seldom conveyed interest in doing so [8].

Recent investigations have documented the presence of focused and intensive MA advertising campaigns that emphasize “zero-premium” benefits while obscuring the limitations of the provider network, the degree to which cost-sharing is involved, and/or possibly onerous prior-authorization rules [9]. These imbalances create a complex environment, in which older adults, particularly those with lower health literacy or digital access, must interpret promotional information that may not align with their care needs or financial circumstances [10]. As an example of this misalignment, a primary driver of MA enrollment, as indicated by focus groups and surveys, is that MA plans advertise lower out-of-pocket costs relative to traditional Medicare [4]. In practice, however, a study by Park et al [4] found that switching from traditional Medicare to MA was associated with increased financial burden among low-income and other vulnerable populations.

Enrollees seeking cost-effective plans that will meet their health care needs are challenged by the fact that available indicators of plan quality may be unreliable. One metric consumers use to decide on a plan is CMS’s star ratings system [11]. Under the Affordable Care Act, the star ratings system was also used as an incentive to MA companies by offering bonus payments to companies that earned high ratings [11]. However, while early evidence demonstrated a correlation between high ratings and higher-quality care and lower disenrollment rates, a 2022 study by Meyers et al [5] found that there was no association between higher star ratings and improved care outcomes. Additional recent evidence suggests that the higher star ratings are associated with the sociodemographic characteristics of enrollees rather than plan performance [11,12]. The ineffectiveness of the star ratings system has prompted numerous stakeholders, including the Medicare Payment Advisory Commission, the plans themselves, and think tanks across the political spectrum, to issue recommendations for fixes [13].

In addition to facing unreliable indicators of quality, MA enrollees are actively persuaded to choose plans contrary to their own financial and medical interests. The marketing of MA products is dominated by private insurers, brokers, and third-party lead generators, whose activities often outpace the public information efforts of Medicare or the State Health Insurance Assistance Programs [14,15]. In part because of the overwhelming number of MA plans, consumers commonly turn to brokers for guidance on selecting an appropriate plan [16]. However, MA plans offer brokers robust monetary incentives to steer enrollees toward more profitable plans [17]. Similar to what occurred involving the ineffectiveness of the star ratings system, various proposals have been set forward to mitigate the tendency for brokers to place their own financial interests above enrollees’ needs, but existing remedies lack efficacy. As an example, State Health Insurance Programs offer free counseling and lack financial incentives to favor plans, but these programs are typically underfunded and rely on volunteers [17].

To date, the peer-reviewed literature provides limited empirical evidence directly evaluating whether Medicare beneficiaries’ selection of MA plans is predominantly driven by media marketing. KFF (formerly the Kaiser Family Foundation) documented that MA dominated open enrollment television advertisements and that these spots frequently emphasized extras (dental, vision, and hearing benefits) and certain “savings,” often without providing core plan details. This demonstrates intensity of exposure but not causation [18,19]. Qualitative, self-reported evidence of influence and confusion comes from focus groups and surveys (KFF, Medicare Rights Center, and Commonwealth Fund), which report that beneficiaries feel overwhelmed by advertisements and outreach. Some beneficiaries say that marketing affects their perceptions and choices: CMS tightened marketing rules after consumer watchdogs submitted complaints about misleading advertisements and highlighted problematic practices. This demonstrated concern but not concern that advertisements are the predominant driver of choice [20]. Costs or premiums, perceived out-of-pocket savings, extra benefits, star ratings, provider networks, and consumer inertia (not reviewing other options) are repeatedly cited in research studies as major factors involved in plan selection. KFF found that approximately 70% of beneficiaries did not compare plans during open enrollment, while other reports point to cost perceptions as a key motivator [21].

Evidence indicates that consumer marketing by health plans influences beneficiary plan enrollment and may tilt offerings toward select populations based on anticipated use and referral patterns. A Commonwealth Fund survey found that nearly half of beneficiaries relied primarily on insurance agents or brokers when choosing a plan, often without verifying whether their preferred clinicians were in-network or whether their current medications were covered [14]. Another national analysis concluded that marketing intensity, rather than objective plan quality, consistently predicted local enrollment growth [22]. Vulnerable groups, including individuals with low incomes and racial or ethnic minority beneficiaries, may be disproportionately affected, as these populations are more likely to depend on television and social media advertising for information [22,23].

Furthermore, once enrolled, Medicare-eligible beneficiaries who selected an MA plan faced significant challenges when attempting to switch to a different MA plan. The vast array of initial choices, along with the complex cognitive processes required for selection, disincentivizes many enrollees from revisiting their choices following initial enrollment [24]. A 2021 study by Rivera-Hernandez et al [24] found a persistent disinclination to switch plans among Medicare-eligible beneficiaries, despite the fact that for many of them, switching plans could result in reduced costs, more preferable provider networks, and/or benefits better suited to their evolving health needs.

Although switching between different MA plans is fraught with difficulties, switching from MA to traditional Medicare is very costly and can even be impossible. This is despite the fact that, as identified in the aforementioned study by Park et al [4], the financial burden from being enrolled in traditional Medicare when compared to MA would be lower for many low-income and otherwise vulnerable populations [17]. With the exception of MA enrollees who live in 4 states with policy protections in place (Connecticut, Maine, Massachusetts, and New York), MA enrollees who wish to return to traditional Medicare can face underwriting restrictions on supplemental “Medigap” policies [8,17]. Consequently, MA beneficiaries seeking to switch to traditional Medicare routinely find that no traditional Medicare plan will issue them a policy, or that the policies offered are prohibitively expensive. This experience is especially common among patients in poor health [17]. Because of these barriers, the annual rate of enrollees switching from MA to traditional Medicare was a mere 1.2% in 2022, down from 5% in 2014 [17]. In effect, enrollment in MA is likely a lifelong and irreversible decision, and enrollees often do not understand the permanence of their commitment [17].

Consequently, MA decision-making reflects a system-level challenge of information asymmetry, where the balance of knowledge and persuasion favors commercial actors over consumers. Recognizing that many patients find decision-making burdensome due to a lack of understanding of Medicare option choices coupled with the economic and health consequences of choosing an appropriate option, misleading marketing practices by MA plans are a cause for concern for a vulnerable population [24].

### Digital Media as a Behavioral Signal

The migration of health plan marketing to digital channels has paralleled broader societal shifts in information-seeking behavior. Older adults increasingly use internet search engines to compare coverage options, locate providers, and investigate benefits. By 2023, nearly 80% of the American population aged 65 to 74 years reported regular internet use, up from 45% in 2013. This digital adoption enables measurement of public attention and interest through digital internet search trace data, including Google Trends, social media analytics, and online advertisement impressions. Google Trends, in particular, offers an accessible, anonymized, and longitudinal indicator of public search activity. It has been validated across domains such as infectious disease surveillance, vaccination uptake, and health service use forecasting [25]. However, it has not been systematically applied to consumer behavior around seeking health insurance, where consumer choices are both time-bound (limited to enrollment periods) and highly sensitive to marketing stimuli.

### Rationale for This Study

The MA market provides an ideal scenario for testing whether online search behavior mirrors consumer decision-making in a regulated insurance environment. The program’s annual enrollment period (AEP), typically from October to December each year, creates a recurring, policy-defined window of heightened consumer engagement. During this interval, advertising expenditures surge, and search engines become the primary conduit for plan comparison and lead generation. Unlike most health policy datasets, Google Trends captures this activity in near real time, allowing researchers and policymakers to observe fluctuations in interest, intensity, and geography. Because near-real-time MA enrollment data are not publicly available to health service researchers or policy analysts outside CMS and participating insurers, population-level search metrics may function as an early warning signal for emerging disparities, misinformation, or potential oversaturation of marketing within specific markets.

Prior work has shown that older adults rarely rely on official online resources such as Medicare when evaluating coverage and instead prefer interpersonal or broker-mediated information sources [23]. Consequently, digital search behavior may represent both direct consumer curiosity and the footprint of marketing reach. Where commercial entities invest heavily in online advertising or search engine optimization, search volumes are expected to rise, even if the quality of information remains uneven. This dynamic creates opportunities for empirical measurement. Correlating search volume with advertising data and enrollment outcomes can reveal how attention translates into action and where policy interventions might strengthen the integrity of consumer choice.

### Contribution to Health Policy Research

From a health systems perspective, the capacity to monitor public interest and marketing saturation in real time is increasingly important for regulatory oversight, equity analysis, and program evaluation. Traditional survey instruments and administrative datasets, while rigorous, often lag behind dynamic market conditions. Applying Google Trends to MA offers a novel, low-cost complement to these tools. If validated, search data could help federal and state agencies identify markets with disproportionate promotional exposure, evaluate the effectiveness of educational campaigns, and target interventions to populations at risk of misleading marketing. Furthermore, integrating behavioral data on internet searchers with existing CMS dashboards could enhance transparency by aligning consumer-level engagement metrics with enrollment trends.

### Objectives

To address these gaps, this study leverages 20 years of US search trend data to examine whether consumer internet behavior reflects the intensity of marketing and enrollment in MA. We pursued three specific objectives: (1) to establish the validity of Google Trends data as a surrogate measure for consumer MA plan selection by demonstrating stable, repeatable seasonality and domain specificity using control terms such as “car insurance” and “life insurance” at national and Designated Market Area (DMA) levels; (2) to quantify the congruency between MA search interest and CMS enrollment data by testing whether search peaks coincide with or precede enrollment surges nationally within a year; and (3) to assess whether local search intensity aligns with advertising exposure by evaluating search behavior as a potential proxy for marketing impact and consumer engagement.

Together, these objectives situate digital trace data within the broader context of health system performance and consumer protection and provide empirical insight into how information environments influence insurance coverage choices among older adults.

## Methods

### Study Design and Analytic Approach

We conducted a retrospective time-series study to examine patterns of consumer internet search behavior related to MA plans in the United States between January 2004 and December 2024. The design integrates publicly available Google Trends data with CMS enrollment statistics and television advertising data obtained from KFF and the Wesleyan Media Project.

The analytic framework adapts and extends the methodology of Szewczyk and Zygmunt [25], who previously validated Google Trends for measuring insurance-related public interest in Europe. This study applies similar time-series decomposition and forecasting techniques, specifically Holt-Winters exponential smoothing and Seasonal Autoregressive Integrated Moving Average (SARIMA) modeling, to the US MA market, where enrollment and marketing cycles are tightly defined by policy.

### Data Sources

#### Google Trends

Google Trends provides deidentified, aggregated indices of search activity within the Google search engine, expressed as relative search volume (RSV) normalized on a 0-100 scale. RSV reflects the proportionate intensity of interest over time within a platform, rather than the absolute search counts or population-level prevalence. A value of 100 indicates the peak popularity of a search term during the specified period and geography, while lower values represent proportionate fractions of that maximum.

Absolute search volumes or population-normalized measures would be preferable but are not available through Google Trends. Because RSV preserves proportional changes within a search term over time, it is well-suited for identifying seasonality, timing, and comovement with enrollment and advertising cycles, which are the primary outcomes of interest.

We queried Google Trends for the following MA-related terms: “Medicare Advantage,” “Medicare Advantage plan(s),” and “Medicare insurance plan(s).” We then combined singular and plural forms into a unified time series to ensure consistent signal strength. After aggregation, the RSV series for each term was linearly rescaled by dividing by its mean within each analysis window. This secondary normalization preserved proportional temporal variation and prevented mechanical scaling artifacts introduced by Google’s 0-100 indexing without affecting seasonal patterns, correlations, or regression estimates. Data were downloaded in CSV format through the Google Trends web interface, using the “Health Insurance” category filter to minimize noise from unrelated topics. To benchmark health-specific patterns, we analyzed 2 control terms, “car insurance” and “life insurance,” using identical procedures.

#### MA Enrollment

Monthly enrollment data were drawn from the CMS Medicare Advantage Enrollment Dashboard, which aggregates plan-level counts of beneficiaries enrolled in MA and MA-Prescription Drug plans. These data provide the official record of plan uptake by month and geography and enable assessment of temporal alignment between search behavior and actual enrollment.

#### Advertising and Marketing Exposure

To evaluate associations between marketing intensity and search behavior, we used data compiled by KFF and the Wesleyan Media Project. This dataset captures the volume, timing, and sponsor characteristics of Medicare-related television advertisements aired across English-language broadcast and national cable networks during the 2022 Medicare Open Enrollment Period. The analytic subset included 1267 unique MA advertisements aired 643,852 times across US DMAs, the nonoverlapping geographic regions that group counties based on television viewing areas.

### Variables and Measures

#### Independent Variable

The primary independent variable was RSV for MA-related terms, representing aggregate online attention to MA.

#### Dependent Variables

Two dependent measures were examined: (1) monthly enrollment changes, defined as the month-over-month difference in CMS MA enrollment counts, capturing active consumer decisions during the AEP, and (2) local advertising exposure, defined as the log-transformed number of MA television advertisement airings per DMA during each 3-month period (preenrollment, enrollment, and postenrollment).

#### Derived Components

Each RSV time series was decomposed into three components using exponential smoothing: (1) level component, defined as the baseline magnitude of search interest; (2) trend component, defined as long-term directional change; and (3) seasonal component, defined as recurrent 12-month fluctuations reflecting cyclical patterns in consumer engagement. This decomposition enabled the detection of stable seasonality and long-run shifts independent of transient noise.

### Bias and Confounding

Several sources of bias were considered. First, sociodemographic and digital access disparities may lead to underrepresentation of older adults or low-income populations in online search data, and changes in Google’s market share over time may affect the representativeness of early RSV values. Accordingly, this study does not assume that Google searches constitute a random sample of all MA-related information seeking, particularly in the mid-2000s, but interprets Google Trends as a within-platform measure of relative attention with greater external validity in later years. Reverse causality is possible if insurers allocate advertising budgets to markets already showing high digital engagement. Finally, unobserved contextual factors, such as local plan availability, historical enrollment patterns, or concurrent media coverage, may confound associations between marketing and search activity. To mitigate these limitations, analyses were restricted to aggregate-level patterns and interpreted descriptively rather than causally.

### Statistical Analysis

#### Time-Series Modeling

We modeled long-term patterns using the Holt-Winters multiplicative model, which is suitable when seasonal amplitude scales with the overall trend. The model decomposes observed values *Y_t_* into level (*L_t_*), trend (*T_t_*), and seasonal (*S_t_*) components, such that

*Y_t_*=(*L_t_* +*T_t_*)×*S_t_*+*ε_t_*

where *ε_t_* denotes the random error term. Model smoothing parameters (α,β,γ) were optimized by minimizing the Akaike information criterion. Forecast accuracy was validated using a SARIMA (1,1,1)(0,1,1) [12] model, with parameters identified via the auto.arima() function in R (version 4.3; R Foundation for Statistical Computing). The close correspondence of seasonal forecasts between models served as a robustness check for structural stability.

#### Correlation With Enrollment

To assess temporal concordance, the MA RSV series was aligned with CMS monthly enrollment data (February 2013-December 2024). Pearson *r* correlations were calculated between the first differences of national enrollment counts—accounting for the cumulative nature of the time series—and contemporaneous RSV values, characterizing month-over-month alignment of search activity and enrollment surges within each calendar month. No lag was applied in this analysis; correlations reflect synchronous, within-month alignment of search behavior and enrollment changes, although future analyses could quantify the extent to which search activity precedes enrollment. Visual overlay plots compared the timing of peaks and troughs and evaluated whether search surges preceded or coincided with enrollment spikes.

#### Association With Advertising Intensity

For DMA-level analyses, three intervals were defined: (1) preenrollment, defined as July to September 2022; (2) enrollment, defined as October to December 2022; and (3) postenrollment, defined as January to March 2023.

A multivariate linear regression model estimated the relationship between log-transformed advertisement airings and enrollment period RSV, controlling for preperiod RSV to adjust for baseline interest. Statistical significance was set at *P*<.05. Heteroscedasticity-robust SEs were used to account for variance instability across markets. All analyses and visualizations were performed in R (version 4.3) using the *forecast*, *ggplot2*, and *tidyverse* packages.

#### Data Access and Linkage

All datasets used were publicly accessible and contained no personally identifiable information: (1) Google Trends, which was accessed via the public Google Trends interface; (2) the CMS Enrollment Dashboard, which was downloaded from the CMS open-data portal; and (3) advertising data, which were obtained through KFF’s public repository in partnership with the Wesleyan Media Project [26,27]. Data from different sources were linked at the aggregate national or DMA level using shared temporal identifiers (month and year).

### Ethical Considerations

No individual-level matching was performed; therefore, institutional review board approval was not required.

## Results

### Objective 1: Establishing the Construct Validity and Seasonality of Google Trends as a Measure of MA Search Behavior

#### Overview

**Figure 1:**
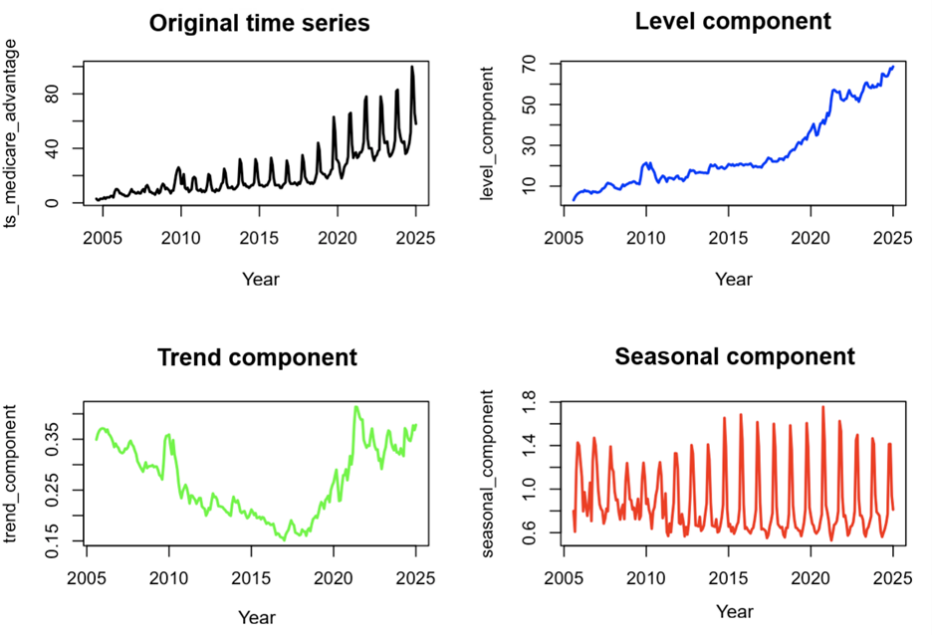
Decomposition plot, “Medicare Advantage.”

Decomposition of the Google Trends time series for “Medicare Advantage” revealed 3 stable components across the 20-year study period (Figure 1).

The level component, representing the seasonally adjusted baseline of search interest, showed a steady rise beginning in 2005, plateauing briefly from 2011 to 2017, and accelerating sharply shortly after 2019. The trend component captures the rate of change of the level; a corresponding decrease pre-2017 reflects a period of slower growth in baseline interest, followed by accelerated growth in later years, producing a U-shaped pattern. Importantly, even when the trend component declines, the underlying trend remains positive and increasing. This confirmed a long-term upward trajectory in search engagement, corresponding to national MA enrollment growth and policy expansion. The seasonal component displayed pronounced cyclical peaks every October-December, coinciding with the CMS AEP, and deep troughs in late spring (April-June).

Model-derived monthly seasonal indices quantified these fluctuations. Search activity was highest in October (1.703) and November (1.641), approximately 70% and 64% above baseline, respectively, and lowest in May (0.718), or 28% below baseline (Table 1).

The full seasonal range (0.718-1.703) represents a 98.5% amplitude difference between trough and peak months, far exceeding that observed for control terms (“car insurance” and “life insurance,” each approximately 22%).

The distribution of RSV (Multimedia Appendix 1) for the term “Medicare Advantage” is right-skewed, with most observations clustered at lower RSV values, and a long upper tail reflecting intermittent periods of heightened search intensity. This distribution suggests substantial temporal variability with occasional high-intensity search periods.

**Table 1:** Seasonal indices for “Medicare Advantage.”

Month	Seasonal index
January	0.913
February	0.890
March	0.856
April	0.800
May	0.718
June	0.745
July	0.791
August	0.846
September	0.943
October	1.703
November	1.641
December	1.154

#### Forecast Validation

**Figure 2:**
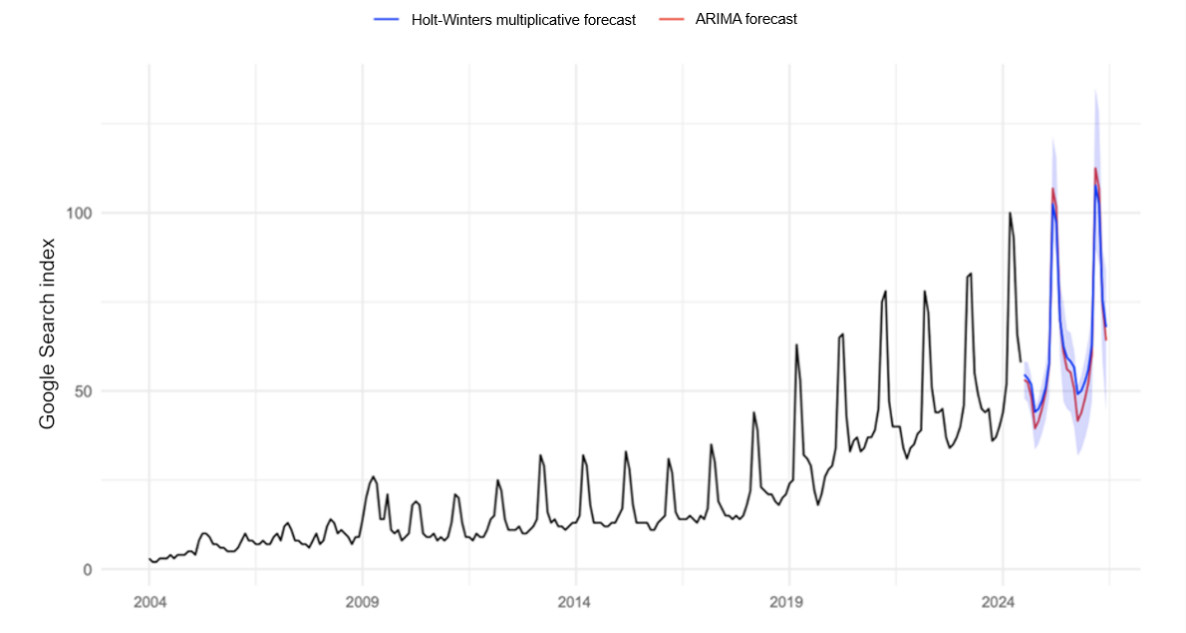
Forecast comparison—Holt-Winters versus ARIMA for “Medicare Advantage.” ARIMA: Autoregressive Integrated Moving Average.

Holt-Winters multiplicative forecasts were compared with a SARIMA (1,1,1)(0,1,1) [12] model to assess structural stability. The 2 models exhibited nearly identical seasonal amplitudes and overlapping 95% CIs, as shown in Figure 2.

**Figure 3:**
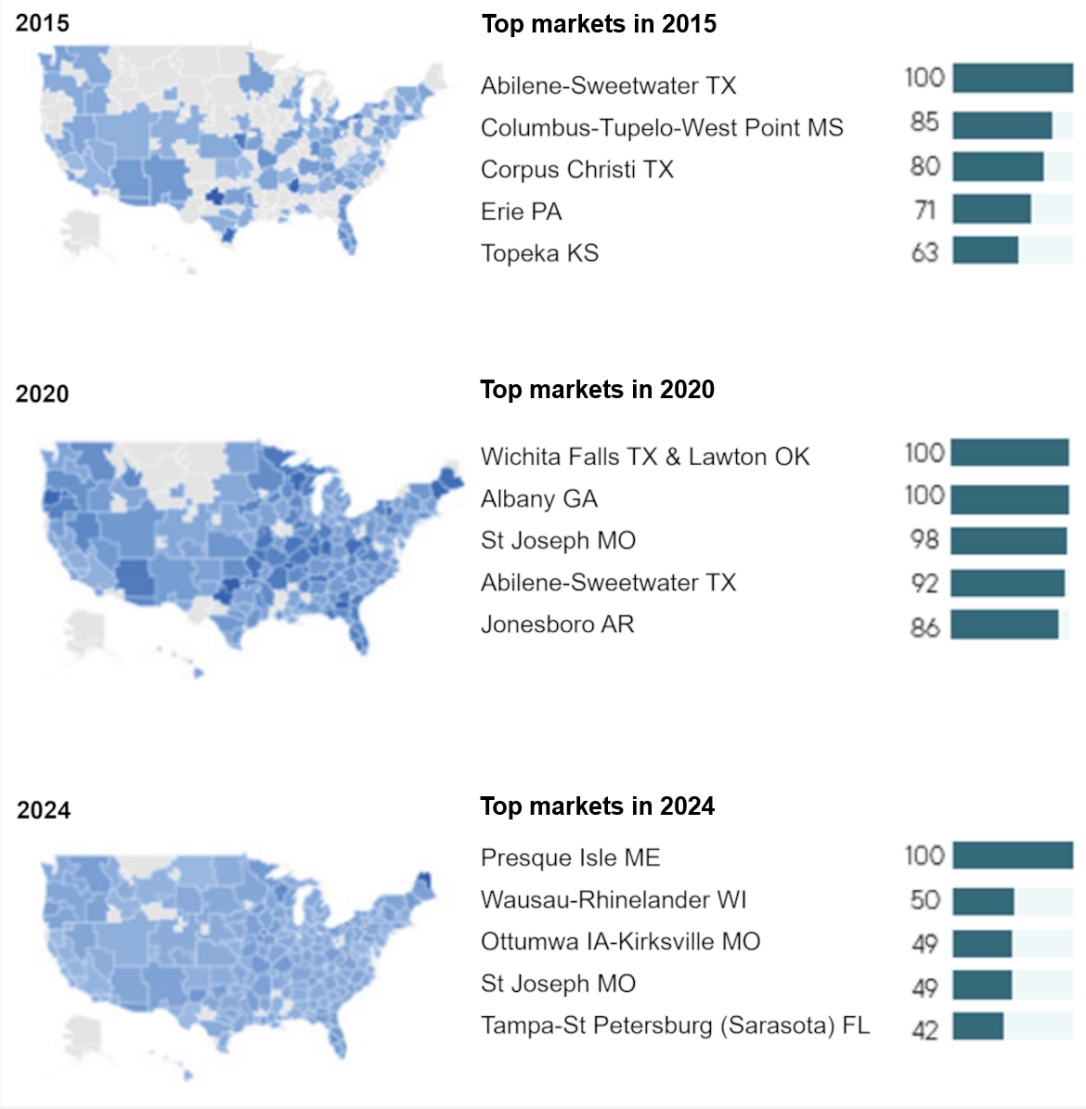
Geographic distribution in 2015, 2020, and 2024.

Root-mean-square-error differences were <5%, confirming robustness and indicating that search activity follows a highly predictable, policy-driven seasonal rhythm rather than random fluctuation. Geospatial visualization exhibited changes in search intensities in markets over time. Between 2015 and 2024, areas of high search concentration expanded from a few southern and midwestern clusters to near national coverage (Figure 3). By 2024, 49 of 50 states demonstrated elevated seasonal search peaks during the AEP, reflecting the diffusion of MA marketing across the United States.

Observationally, large metropolitan DMAs displayed both higher absolute advertisement volume and greater elasticity of search response, consistent with the idea that commercial marketing ecosystems amplify digital visibility in densely populated regions. These patterns are descriptive and not formally adjusted for population and should therefore be interpreted cautiously.

### Objective 2: Temporal Alignment Between MA Search Activity and CMS Enrollment Patterns

#### Overview

**Figure 4:**
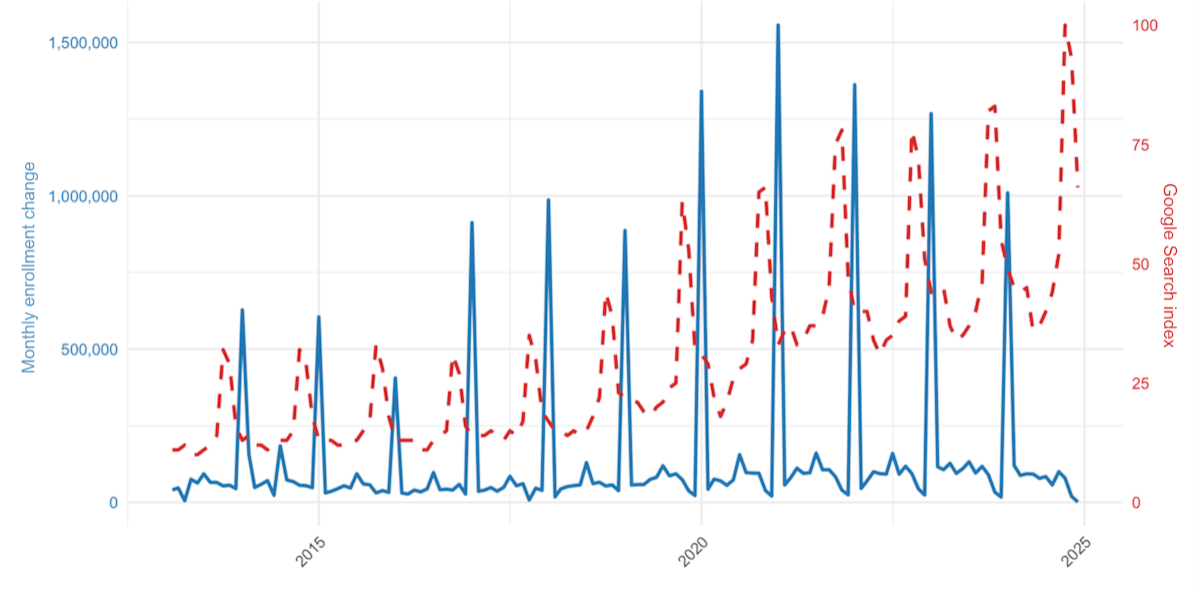
Medicare Advantage enrollment versus Google searches. Source: CMS and Google Trends.

Figure 4 overlays national MA search indices with monthly enrollment changes from CMS between February 2013 and December 2024. Search interest rose gradually beginning in late summer, reached its peak in October-November, and declined rapidly after December, mirroring the enrollment surge that occurs during the AEP. Month-over-month changes in enrollment lagged search interest by roughly 2 to 4 weeks, suggesting that search behavior precedes and possibly signals subsequent plan selection activity.

#### Correlation Analysis

The relationship between monthly RSV values and new MA enrollments was statistically correlated and consistent across years. The mean Pearson correlation coefficient (*r*) between first-difference search and enrollment series was 0.72 (*P*<.001) at the national level, indicating a robust temporal alignment between digital engagement and actual plan uptake.

This correlation remained significant when controlling for overall time trend, supporting the interpretation of search interest as a leading indicator of enrollment behavior rather than a mere reflection of cumulative program growth. Over time, both baseline and peak RSV values increased after 2019, which coincides with MA market penetration rates, intensified health plan marketing plans, and postpandemic digital health adoption rates among Medicare beneficiaries. The rising baseline indicates that consumer attention to MA is becoming sustained year-round and is not limited solely to open enrollment periods.

### Objective 3: Evaluating Local Search Intensity to MA Advertising Exposure and Market Engagement

**Figure 5:**
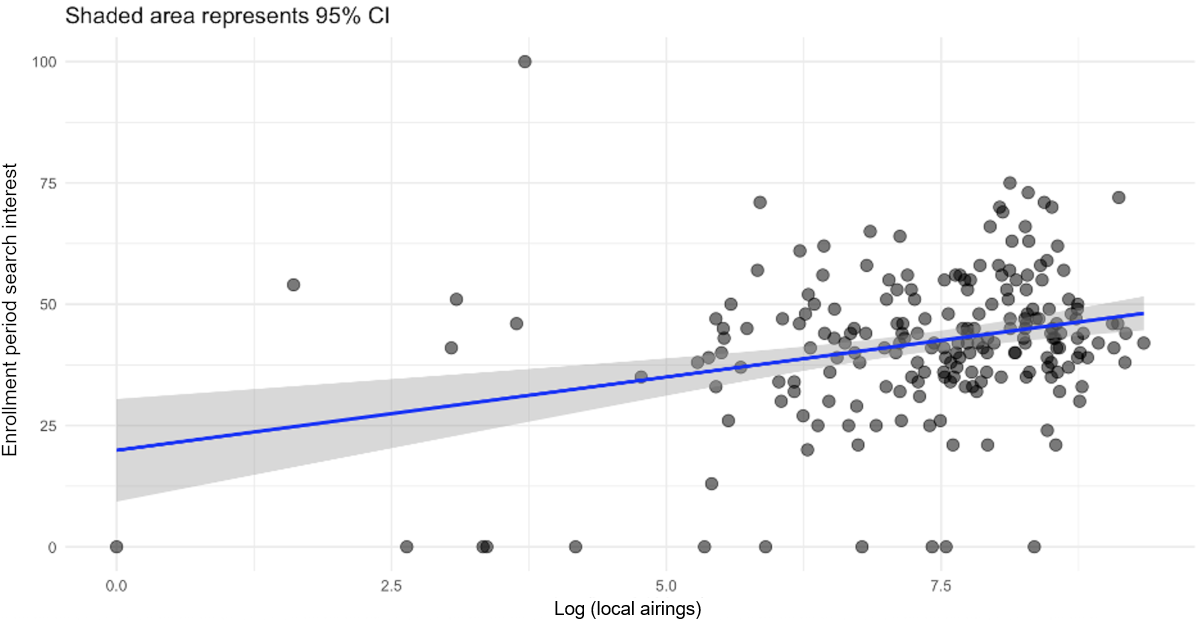
Local advertisement airings versus enrollment period searches.

To examine whether marketing exposure predicts consumer search engagement, Google Trends data for 210 US DMAs were linked with local television advertising volumes during the 2022-2023 enrollment cycle (Figure 5). The following three temporal windows were analyzed: (1) preenrollment, defined as July to September 2022; (2) enrollment, defined as October to December 2022; and (3) postenrollment, defined as January to March 2023.

Across DMAs, the log-transformed number of advertisement airings was positively associated with enrollment period RSV for “Medicare Advantage” after adjusting for baseline preperiod interest (*β*=.42, SE=0.07; *P*<.001). The model explained 48% of the variance (adjusted *R*^2^=0.48) in local search activity, indicating that higher advertising volume corresponded to greater digital engagement.

### Model Performance and Comparative Trends

Comparative analyses confirmed that MA search dynamics differ markedly from search dynamics in other insurance sectors. For the control terms “car insurance” and “life insurance,” seasonal ranges were limited (0.881-1.075 and 0.898-1.095, respectively), with no consistent October-December peaks. These findings underscore the policy-specific cyclicality of MA search behavior, which is tied directly to enrollment rules rather than to general consumer finance cycles. Overall model diagnostics demonstrated minimal autocorrelation in residuals (Durbin-Watson statistics between 1.9 and 2.1), indicating good model fit. The Holt-Winters and SARIMA forecasts both projected continued baseline growth through 2025, albeit with possible plateauing of seasonal amplitude, suggesting a maturing yet persistently digital consumer market.

### Summary of Principal Findings

MA-related search activity demonstrates a pronounced and highly stable seasonal pattern, characterized by consistent peaks in October through November and troughs in late spring across nearly 2 decades, indicating durable, repeatable consumer attention cycles. Temporal alignment analyses further show that fluctuations in search interest closely track, and often precede, changes in enrollment volume, supporting the use of search behavior as a contemporaneous indicator of consumer engagement rather than a causal driver of plan selection. At the market level, regional variation in search intensity is strongly associated with local advertising exposure, suggesting that marketing activity is reflected in measurable online information-seeking behavior. Finally, the geographic footprint of high-intensity search markets has expanded substantially since 2019, consistent with the broader diffusion of MA offerings, increased digital marketing penetration, and growing reliance on online information sources among beneficiaries. Taken together, these findings indicate that Google Trends may provide a reliable, near-real-time signal of consumer awareness and marketing saturation within the MA program.

## Discussion

### Principal Findings

This study provides new evidence that consumer internet search data can serve as a reliable proxy for public engagement and marketing exposure in the US MA market. Using 20 years of Google Trends data linked with enrollment and advertising metrics, we found that MA-related search activity follows a highly predictable seasonal rhythm aligned with the CMS AEP. Peaks in search activity preceded or coincided with enrollment surges, and local advertising intensity was significantly associated with greater search interest at the market level. Together, these findings demonstrate that internet search patterns reflect both policy-driven enrollment behavior and commercial marketing stimuli, establishing Google Trends as a valid real-time indicator of consumer attention within a federally regulated insurance program.

### Relationship to Previous Literature

Our findings extend previous research on information sources and decision-making among Medicare beneficiaries. Rivera-Hernandez et al [23] reported that few Medicare-eligible beneficiaries rely on Medicare when choosing plans and instead depend on brokers, family, or television advertising, sources that often present incomplete or biased information. Likewise, analyses by the Commonwealth Fund found that marketing substantially shapes plan choice, sometimes at the expense of quality or cost considerations [9,10,14,15,22]. This study quantifies this dynamic at scale. By showing that search volumes increase in direct proportion to advertising exposure, we provide population-level evidence that marketing influences digital information-seeking behavior, which in turn aligns with actual enrollment activity. Beyond marketing-related dynamics, these findings are consistent with prior health services research, showing that online search trends covary with health care use patterns, such as influenza surveillance and cancer screening behavior. By extending this framework to health insurance, we can use digital trace data to capture policy-relevant consumer behavior, while previously we had to rely on delayed administrative reports or costly surveys. This work of validating search-based surveillance for MA bridges public health informatics and health policy evaluation and illustrates how search behavior data can illuminate consumer responses to regulatory environments.

### Interpretation and Policy Implications

The consistency of the observed patterns underscores the extent to which MA enrollment behavior is structured by the patterns of open season for health plan issuances but is amplified by commercial marketing ecosystems. The October-December peaks correspond precisely to the AEP, a period during which insurers and brokers intensify advertising. The significant year-round rise in baseline search volume after 2019 coincides with the expansion of MA benefits, the proliferation of “zero-premium” messaging, and increased digital adoption among older adults during the COVID-19 pandemic [9].

From a policy perspective, these results highlight an urgent need to modernize consumer protection and information equity strategies. MA marketing remains governed largely by post-hoc compliance audits rather than real-time monitoring. Digital analytics such as Google Trends could provide regulators and researchers with early warning signals of excessive or misleading marketing concentration in specific markets. Because search data are publicly available, inexpensive, and updated daily, they could complement CMS’s existing oversight mechanisms by flagging anomalies that merit deeper investigation or by prompting targeted education campaigns through trusted channels.

For policymakers and advocacy organizations, real-time monitoring of search data could also inform equity-oriented interventions or inform consumer regulatory agencies about marketplace forces that influence consumer choice. Markets with high search intensity but low access to unbiased counseling (eg, low State Health Insurance Assistance Program capacity) may face a greater risk of misinformed enrollment. Similarly, communities with limited broadband access might be underrepresented in digital data, yet simultaneously more vulnerable to aggressive offline marketing. Digital surveillance integrated with demographic and geographic indicators could help identify such disparities and support targeted outreach to underserved groups.

### Internet Search Behavior and Systems Perspectives

At the level of internet search behavior, the observed correlations suggest that search behavior represents both curiosity and intent, a stage in the decision pathway during which information asymmetry is most consequential. Advertising increases awareness, but it can also distort perceived value; beneficiaries often focus on salient marketing claims rather than on objective plan metrics [10,22]. The association between advertisement volume and search engagement reflects not only exposure to the marketing material but also heightened consumer attention. From a systems viewpoint, these digital patterns offer a window into how a publicly funded program operates within a private marketing infrastructure. The convergence of policy development, commercial incentives, and consumer online behavior underscores the hybrid nature of the MA marketplace—public in financing but market-driven in communication.

### Comparison With Other Insurance Sectors

Control analyses that we conducted using the terms “car insurance” and “life insurance” demonstrated minimal seasonality and weak correlations with advertising cycles and confirmed that the statistically correlated periodicity observed for MA is not a generic feature of consumer finance behavior. MA decisions, unlike decisions involving voluntary consumer products, are concentrated within a policy-defined enrollment window that produces sharp, predictable peaks. This specificity reinforces the methodological validity of using search data for time-bounded health policy phenomena where external deadlines and incentives govern public engagement.

### Strengths and Limitations

This study’s principal strength is the integration of 3 independent data sources—search behavior, enrollment statistics, and advertising volume—over a period of 2 decades. This triangulation provides convergent evidence that online engagement may both precede and reflect measurable policy outcomes. The methodological framework is transparent, reproducible, and adaptable to other contexts, including Medicaid redeterminations and Affordable Care Act Health Insurance Marketplace enrollments.

Several limitations of this Google Trends–based analysis should be acknowledged. First, causality cannot be inferred. Advertising may follow rather than drive search interest, and both may be influenced by unobserved regional characteristics. Second, Google Trends indices are relative measures, normalized within each time window, thus limiting comparability of absolute magnitudes across years or geographies. Third, because the AEP is shared across MA and stand-alone Medicare Part D plans, some degree of overlap in beneficiary search behavior is possible; although we selected search terms that explicitly reference MA or bundled plan types, residual contamination from Part D–only searches cannot be fully excluded. Fourth, digital divide bias persists; older adults with lower digital literacy or broadband access may be underrepresented, and this could skew observed engagement toward more connected populations. Finally, the analysis captures exposure and attention but does not capture comprehension—high search volume does not guarantee informed decision-making. These caveats underscore the need to interpret digital trace data as complementary, not substitute, evidence of consumer behavior.

### Implications for Future Research

Future studies could integrate search data with plan quality ratings, demographic indicators, and enrollment outcomes to identify which populations are most influenced by marketing and which sources (television, online, and broker outreach) drive the greatest behavior response. Mixed methods designs that incorporate surveys or qualitative interviews could validate whether search interest translates to understanding or merely reflects exposure. Additionally, natural experiments—such as policy changes that restrict certain types of advertisements—could test how digital engagement responds to regulatory interventions. Such experiments could offer a mechanism for rapid policy evaluation.

By documenting the alignment among marketing exposure, search engagement, and enrollment, this study provides empirical evidence that digital surveillance tools can enhance transparency and accountability in publicly financed health programs. Google Trends offers a low-cost, scalable complement to traditional data systems and is capable of identifying emerging disparities and informing responsive oversight. As MA enrollment continues to expand, leveraging consumer internet search behavior data could help policymakers ensure that consumer choice involving MA can be informed, equitable, and aligned with the goals of the Medicare program.

### Conclusions

This study demonstrates that publicly available Google Trends data can provide valid, near-real-time indicators of consumer marketing exposure to and engagement in the MA market. Over 2 decades, MA-related search activity has exhibited a consistent, policy-defined seasonal rhythm aligned with the AEP and a statistical correlation with both advertising intensity and enrollment behavior. These findings confirm that digital trace data capture meaningful patterns of public attention within a highly marketed federal insurance program.

From a health systems perspective, this approach offers a scalable and low-cost tool for continuous monitoring of consumer information environments, one that complements traditional surveys and administrative datasets that are slower to update. Search trend surveillance could support regulators, researchers, and policymakers in identifying markets characterized by disproportionate marketing activity or informational inequities and help to inform more responsive outreach and oversight. As digital engagement continues to shape how older adults evaluate and access health coverage, integrating consumer internet search behavior analytics into policy evaluation will be critical for promoting transparency, equity, and informed consumer choice in Medicare and beyond.

## Data Availability

The datasets generated or analyzed during this study are available from the corresponding author on reasonable request.

## Appendix

Multimedia Appendix 1 Distribution of relative search volume for "Medicare Advantage" search term.

## Abbreviations

AEP: annual enrollment period
CMS: Centers for Medicare & Medicaid Services
DMA: Designated Market Area
KFF: formerly Kaiser Family Foundation
MA: Medicare Advantage
RSV: relative search volume
SARIMA: Seasonal Autoregressive Integrated Moving Average
