# Supplementary figures and images for: Quantifying Consumer Interest in Medicare Advantage: Development and Usability Study Using Google Trends Data
Source: JMIR Ment Health. 2026 Mar 27;13:e89355. doi: 10.2196/89355 (PMC13069369; doi:10.2196/89355)

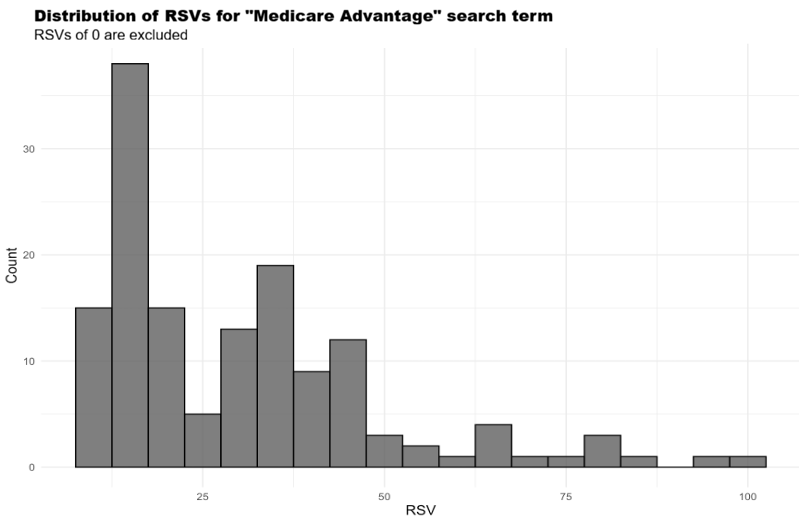

Supplement: Multimedia Appendix 1 [file mental_v13i1e89355_app1.png]
